# The Second-Look Procedure for Transoral Videolaryngoscopic Surgery for T1 and T2 Laryngeal, Oropharyngeal, and Hypopharyngeal Cancer Patients: Protocol for a Nonrandomized Clinical Trial

**DOI:** 10.2196/resprot.8907

**Published:** 2017-12-05

**Authors:** Goshi Nishimura, Daisuke Sano, Kenichiro Yabuki, Yasuhiro Arai, Yoshihiro Chiba, Teruhiko Tanabe, Nobuhiko Oridate

**Affiliations:** ^1^ Department of Otorhinolaryngology, Head and Neck Surgery School of Medicine Yokohama City University Yokohama Japan

**Keywords:** early T stage head and neck cancer, transoral surgery, second-look procedure

## Abstract

**Background:**

Transoral videolaryngoscopic surgery (TOVS) has been widely applied for early T stage head and neck cancer. The resection is performed with a minimum safety margin for function preservation under a limited surgical field of view, making it difficult to be certain of complete resection.

**Objective:**

Our aim is the evaluation of the completeness of resection by initial TOVS resection, and the possibility of primary control by TOVS alone, allowing for repeat procedures for function preserving treatment in early T stage laryngeal, oropharyngeal, and hypopharyngeal cancer patients.

**Methods:**

Patients are treated by TOVS for the primary site with or without neck dissection. Patients are divided in two groups based on the results of the pathological evaluation of the surgical specimen; the control group in which the resection is considered to be complete, and the intervention (second-look procedure) group in which incomplete tumor resection is suspected. The predictive factors for the possibility of complete resection by TOVS will then be analyzed.

**Results:**

Patient enrollment started on January 1, 2014, and closed on March 31, 2016, with 54 patients. The control group consists of 27 patients, the intervention group is 21 patients, and 6 patients were excluded. There were no clinical differences between the control and intervention groups. The observation period will end on December 31, 2018.

**Conclusions:**

TOVS has potential for both definitive resection and function preservation with minimal invasiveness. Identifying the limitations of TOVS is beneficial to ensure accurate treatment selection in early T stage head and neck cancer patients.

**Trial Registration:**

UMIN Clinical Trials Registry: UMIN000012485; https://upload.umin.ac.jp/cgi-open-bin/ctr_e/ctr_view.cgi? recptno=R000014472 (Archived by WebCite at http://www.webcitation.org/6v1b741Iw)

## Introduction

The treatment of head and neck cancer demands attention be paid both to definitive curability and function preservation, for example, phonation and deglutition. Although most head and neck cancer patients indicate advanced stage at the first visit, the number of early T stage patients has been increasing because of the development of diagnostic instruments, such as positron emission tomography-computed tomography (PET-CT) [1] and narrow band imaging (NBI) endoscopy [2], as well as greater awareness of head and neck cancer among doctors in other fields [3]. With the development of surgical support instruments, there have been many innovations in minimally invasive surgery for the treatment of head and neck cancer, especially with regard to definitive surgery for early T stage patients [4-8]. The local or regional recurrence rates are 13.4% [9] for transoral surgery (TOS) and 24.7% [10] for (chemo)radiotherapy in early T stage laryngeal cancer, 14.4% [11] and 22.8% [12] in oropharyngeal cancer, and 25.4% [13] and 30.2% [14] in hypopharyngeal cancer, respectively. Although these data are from a single institutional retrospective study, TOS has been considered to provide equivalent outcomes to conventional (chemo)radiotherapy or open surgery (eg, total or partial laryngectomy, pharyngectomy [4,6,15,16]). In our department, transoral videolaryngoscopic surgery (TOVS) [6] has been applied to T1 and T2 laryngeal, oropharyngeal, and hypopharyngeal cancer as a local surgery with or without neck dissection. We believe that one of the main problems associated with TOVS is the issue of completeness of resection due to the limited field of view and lack of maneuverability, as well as the minimum safety margin required for maximum function preservation. In this study, we sought to identify the completeness or limitation of TOVS through the application of the second-look procedure in cases of suspected incomplete tumor resection.

## Methods

### Study Setting

This parallel two-arm open-label nonrandomized trial is taking place at a single institute (ie, an academic hospital).

### Endpoints

The primary endpoints are the evaluation of the completeness of resection by the initial TOVS resection, and the possibility of primary control by TOVS alone, allowing for repeat procedures of salvage surgery in cases of suspected incomplete tumor resection at the initial TOVS. The secondary endpoints are overall survival, disease free survival, and function preserving survival.

### Eligibility Criteria

Head and neck cancer is classified according to the 7^th^edition of the TNM classification [17]. Clinical T1 and T2 stage laryngeal, oropharyngeal, and hypopharyngeal carcinoma patients are enrolled.

#### Inclusion Criteria

Prior to enrollment in this trial, the patients must meet all of the following criteria: pathologically proven carcinoma; primary tumor located in the larynx, oropharynx, or hypopharynx; cT1 and cT2 tumor on visual and endoscopic examinations and imaging examinations (eg, CT and/or magnetic resonance imaging [MRI]); cN stage evaluated by ultrasonic echo [US] and/or PET-CT); primary site assessed as resectable by TOVS and regional lymph node by neck dissection on CT, MRI, and/or US; no distant metastasis on PET-CT (cM0); no prior treatment for any head and neck cancers; aged over 20 years old (regarded as a legal adult in Japan); performance status 0-2 on Eastern Cooperative Oncology Group (ECOG) criteria; sufficient general condition for the operation under general anesthesia; and provision of written informed consent.

#### Exclusion Criteria

Prior to enrollment in this trial, the patients must not have incurable synchronous malignancies, priority systemic diseases, nor refuse to undergo the second-look procedure.

Patients who had treatment history in the head and neck are excluded because of the possibility of scar formation that can influence submucosal resection by TOVS. The patients who had previous malignancy are included in case these diseases have been cured or well controlled (maintaining complete response condition).

### Enrollment

Patient enrollment started on January 1, 2014, for a scheduled period of 2 years. Ethics approval for the study was obtained from Yokohama City University Institutional Review Board (#B131107003). Written informed consent was obtained from the participants in the study and for publication of their data.

### Treatment Methods

#### Surgery

Primary resection is performed using the TOVS technique. The mucosal lesion is confirmed by NBI endoscope and Lugol’s solution dyeing. The horizontal safety margin is set at 1-3 mm from the border of the lesion. The vertical resection is performed in the submucosal layer. After resection, rapid pathological margin examination is undertaken for the horizontal and vertical sections. In cases with positive margins as assessed by rapid pathological examination, additional resection is performed until a negative margin is confirmed. The resected specimen is stretched on a cork board to clarify directions and fixed with formalin for permanent pathological diagnosis. The wound is covered with a polyglycolic acid sheet for oropharyngeal and hypopharyngeal cancer patients. In node positive patients, neck dissection is performed at the same time.

#### Control Arm

The patients in whom the resection is assessed as complete with negative margins by both rapid and permanent pathological examinations receive no additional therapy for local control.

#### Intervention (Second-Look Procedure) Arm

The patients in whom complete resection is not unexpectedly confirmed by permanent pathological examination (ie, the presence of positive or close margins) are entered into the intervention arm. Approximately 2-3 months after the first TOVS operation, the second-look procedure (re-TOVS) is performed for these patients.

**Figure 1:**
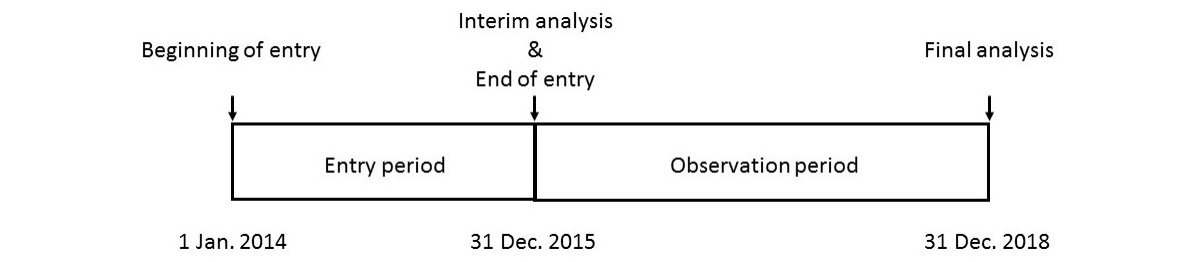
Study schedule.

The period between first and second TOVS are set as wound repair time for precise observation at the second-look TOVS, and close follow-up is applied during the period for these patients. During the second-look procedure, precise observation by high-vision endoscope, pathological examination from the resected primary site, and additional resection for the tumor remnant are performed.

In cases where the additional resection cannot be completed using the TOVS technique, open surgery and/or (chemo)radiotherapy are administered as alternative definitive therapy.

#### Follow-Up

All patients are followed up for at least 5 years after patient accrual has been completed. Visual and endoscopic observations of the primary site are performed every month for the first and second years, and every 2-3 months from the third to fifth years. Enhanced cervical CT and/or US for the primary site and regional lymph nodes is performed every 3-6 months for the first and second years, and every 6-12 months from the third to fifth years. PET-CT is performed every year for the first and second years, and enhanced whole body CT is performed every year from the third to fifth years for the evaluation of distant metastasis.

### Scheduled Analysis

Target sample size is set at 40-60 patients, based on the average patient number (20-30 patients per year) in our institution and the entry period of 2 years. We plan an interim analysis at the end of the entry period. If the number of patients enrolled has reached the target number at the time of the interim analysis, enrollment will cease. If not, enrollment will be extended until at least 40 patients are enrolled. The final analysis is to be performed 5 years after the first entry. The expected schedule is shown in Figure 1.

### Statistical Analysis

For univariate and multivariate comparisons, Fisher’s exact test and a Cox proportional hazards model are used, respectively. The outcome variables are completeness of the first TOVS resection, the possibility of local control by TOVS, and survival. The predictive variables are clinical characteristics (ie, age, sex, primary site, primary subsite, TN stage, tumor shape, enhanced CT observation), pathological characteristics (ie, histological type, differentiation, margin study, lymphatic invasion, vascular invasion, nerve invasion), and others (eg, tumor location in glottic cancer and human papilloma virus infection in oropharyngeal cancer). These predictive variables emerge after the initial TOVS. We therefore expect them to be factors predicting outcome.

## Results

### Trial Status

UMIN Clinical Trials Registry (UMIN000012485) was completed December 14, 2013. Patient enrollment started January 1, 2014, and enrollment closed March 31, 2016, with 54 patients. The observation period will end December 31, 2018.

### Patient Characteristics

Patient enrollment began January 1, 2014, and closed March 31, 2016, with 54 patients (Table 1). We placed 27 patients into the control group and 21 patients into the intervention group. Six patients were excluded based on the following: 2 patients had past treatment history in the head and neck, 2 patients had incurable synchronous malignancies, and 2 patients rejected the second-look procedure.

**Table 1:** Patient characteristics.

			Control group (n=27)		Intervention group (n=21)	
Age in years, range (median)			48-85 (67)		55-84 (71)	
**Sex, n**						
	Male		25		18	
	Female		2		3	
**Primary site, n**						
	Larynx		9		5	
	Oropharynx		10		4	
	Hypopharynx		8		12	
**TN stage, n**						
	**T**					
		Tis		4		0
		T1		12		7
		T2		11		14
	**N**					
		N0		22		21
		N1		1		0
		N2		4		0
		N3		0		0
**Pathology, n**						
	Squamous cell carcinoma		26		21	
	Spindle cell carcinoma		1		0	

## Discussion

### Principal Findings

TOS is considered to be a definitive therapy, comparable to radiotherapy or open surgery, for early T stage laryngeal, oropharyngeal, and hypopharyngeal cancer consistent with organ and function preservation. In the case of early T stage with node-positive patients, TOS is performed with neck dissection. TOS includes TOVS [6,8,16,17], transoral LASER microsurgery (TLM) [9,13,15], transoral robotic surgery (TORS) [4], and endoscopic laryngo-pharyngeal surgery (ELPS) [5,7]. There are advantages and disadvantages associated with all these surgical procedures. Endoscope-assisted surgery (eg, TOVS, TORS, and ELPS) affords precise views that help resection be completed with minimal invasiveness [4-8,16,17]. Straight operation field surgery (eg, TOVS and TLM) provides easy maneuverability in a similar manner to laryngo-microsurgery techniques [6,8,9,13,15-17], while TORS allows a better view and more accurate procedure, especially when working in less accessible areas [4]. Among these techniques, we mainly choose TOVS, as our priorities are precise endoscopic observation and easy access to the target region [6,8,16,17]. On the other hand, one of the common drawbacks to these techniques is the limited view and working field [4-9,13,15-17].

TOVS aspires to definitive resection with minimum safety margins that may contribute to organ and function preservation. However, such an approach increases the risk of remnant tumor. As complete resection is confirmed by pathological evaluation, the actual decision is difficult because the surgical margin is kept to a minimum and the specimen tends to shrink during electrical coagulation and formalin fixation. The can lead to difficulties in identifying positive or close margins. Salvage treatment for remnant and recurrent tumors at the primary site differs by institution. The salvage treatment is selected among a number of options including re-TOS, radical dissection (eg, total or partial laryngectomy and pharyngectomy), and (chemo)radiotherapy, depending on the recurrence status [7,9,18,19]. Radical dissection is considered as the most reliable and effective treatment method, but results in some loss of function (eg, aphonia, dysarthria, and dysphagia). Although (chemo)radiotherapy can preserve the organs, sensory torpor and/or radiation scars cause functional disorders (eg, hoarseness and dysphagia [20,21]). Moreover, radical dissection after (chemo)radiotherapy failure indicates a high incidence of postoperative complications [22]. Taken together, the drawbacks associated with radical dissection and/or (chemo)radiotherapy and the concept of function preservation that underpins TOS lead us to select re-TOVS as our first choice in salvage treatment.

### Limitations

There potential limitations to our study. Research is taking place at only a single institute, and recruited numbers are low. This small sample size may affect the generalizability of results.

### Conclusions

Early detection of remnant and recurrent tumors is necessary when considering re-TOVS. Recurrence after complete resection is stochastic, and it is considered impossible to absolutely prevent such recurrence. However, we think that second-look TOVS can allow disease control by use of the TOVS technique with function preservation. We hypothesize that one of the causes of remnant tumor is the limited observation and resection that follows from restricted field of view and maneuverability. Second-look TOVS is adapted for high-risk patients with remnant tumor, and precise observation and/or biopsy for confirmation of a pathological negative status is performed for any actual cases of no remnant tumor. Additional resection is performed for truly remnant cases. We hope to identify the predictive factors for complete or incomplete resection by TOVS.

## Abbreviations

ECOG: Eastern Cooperative Oncology Group
ELPS: endoscopic laryngo-pharyngeal surgery
MRI: magnetic resonance imaging
NBI: narrow band imaging
PET-CT: positron emission tomography-computed tomography
TLM: transoral laser microsurgery
TORS: transoral robotic surgery
TOS: transoral surgery
TOVS: transoral videolaryngoscopic surgery
US: ultrasonic echo
